# Saffron, a Potential Bridge between Nutrition and Disease Therapeutics: Global Health Challenges and Therapeutic Opportunities

**DOI:** 10.3390/plants13111467

**Published:** 2024-05-25

**Authors:** Rakeeb Ahmad Mir, Anshika Tyagi, Sofi Javed Hussain, Mohammed A. Almalki, Mohammad Tarique Zeyad, Rupesh Deshmukh, Sajad Ali

**Affiliations:** 1 Department of Biotechnology, School of Life Sciences, Central University of Kashmir, Ganderbal 191201, India; 2 Department of Biotechnology, Yeungnam University, Gyeongsan 38541, Republic of Korea; tyagi.anshika9@gmail.com; 3 Department of Botany, Central University of Kashmir, Ganderbal 191201, India; sjavaidjh@gmail.com; 4 Department of Biological Sciences, College of Science, King Faisal University, Al-Ahsa 31982, Saudi Arabia; 5 Department of Agricultural Microbiology, Faculty of Agriculture Sciences, Aligarh Muslim University, Aligarh 202002, India; mohd.zeyad@gmail.com; 6 Department of Biotechnology, Central University of Haryana, Mahendragarh 123031, India; rupesh0deshmukh@gmail.com

**Keywords:** saffron, crocins, crocetin, safranal, picrocrocin, therapeutics, antimicrobial, anti-cancer, nutrients

## Abstract

Plants are an important source of essential bioactive compounds that not only have a beneficial role in human health and nutrition but also act as drivers for shaping gut microbiome. However, the mechanism of their functional attributes is not fully understood despite their significance. One such important plant is *Crocus sativus*, also known as saffron, which possesses huge medicinal, nutritional, and industrial applications like food and cosmetics. The importance of this plant is grossly attributed to its incredible bioactive constituents such as crocins, crocetin, safranal, picrocrocin, and glycosides. These bioactive compounds possess a wide range of therapeutic activities against multiple human ailments. Since a huge number of studies have revealed negative unwanted side effects of modern-day drugs, the scientific communities at the global level are investigating a large number of medicinal plants to explore natural products as the best alternatives. Taken into consideration, the available research findings indicate that saffron has a huge scope to be further explored to establish alternative natural-product-based drugs for health benefits. In this review, we are providing an update on the role of bioactive compounds of saffron as therapeutic agents (human disorders and antimicrobial activity) and its nutritional values. We also highlighted the role of omics and metabolic engineering tools for increasing the content of key saffron bioactive molecules for its mass production. Finally, pre-clinical and clinical studies seem to be necessary to establish its therapeutic potential against human diseases.

## 1. Introduction

Plants have significant metabolic diversity, which are essential for addressing many of the world’s current challenges, such as drug discovery, food security, and ecosystem functioning. Owing to their inimitable biological attributes and health benefits, the identification of novel plant-based bioactive compounds has received great attention among scientists to further explore their biochemical mechanisms. For centuries, traditional remedies have relied on plant-based natural compounds to cure a variety of ailments. Despite the prevalent dominant use of chemically synthesized drugs to treat human diseases, the application of bioactive compounds derived from medicinal plants is also the center of attention due to their fewer side effects [1]. Other reasons for the use of natural compounds include being readily available, being cost-effective, and having very few side effects [2]. Currently, antimicrobial resistance has emerged as a major threat to humans due to the rapid development of antimicrobial resistance (AMR) infection and a lack of new antimicrobial agents. The World Health Organization (WHO) has reported that AMR will lead to high mortality in humans by 2050. Therefore, the identification of novel plant-based bioactive compounds is one of the viable strategies to tackle this problem. Plant-based bioactive molecules (capsaicin, curcumin, resveratrol, catechin, lignans, quercetin, etc.) have received a lot of attention in recent years due to their wide range of pharmacological functions and biological activities, including antioxidant, antimicrobial, anti-inflammatory, anti-stress, and anti-tumor ones, as well as lowering blood glucose and lipids and improving insulin sensitivity [3]. This further supports the notion that plant metabolites or their structural analogues are key for the development of future drugs.

**Figure 1:**
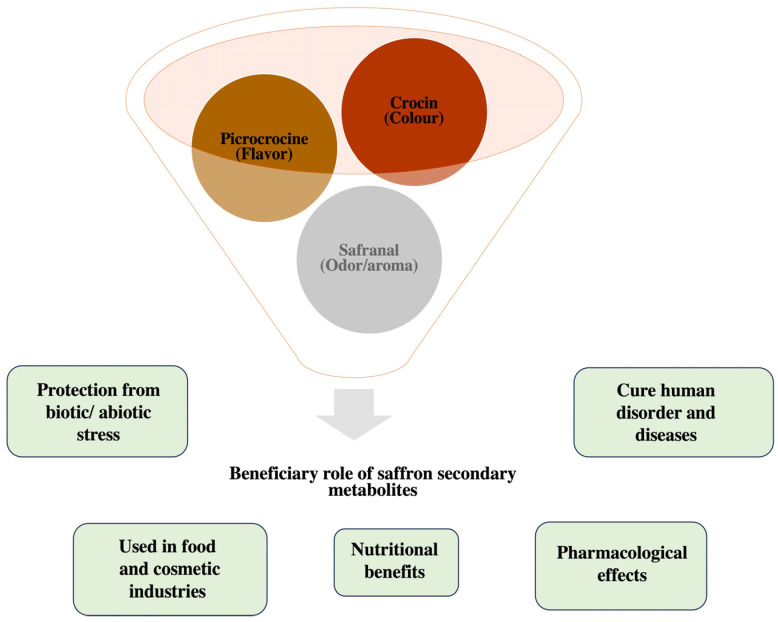
An illustration of the multifaceted role of saffron bioactive compounds.

*Crocus sativus* is a medicinal plant with nutritional and medicinal importance. It is a valuable cash crop grown throughout Europe, the Mediterranean, and central Asia because of its widespread applications as a spice, colorant, and medication [4]. Saffron belongs in the Iridaceae family, which consists of 100 seasonal and perennial species that are cultivated from corm. It is a sterile triploid species (2n = 3x = 24) with a genome size of 3.01 GB. Owing to its wide application in food and cosmetics, it is considered as the world’s most expensive spice, also known as the Golden Condiment. It has been reported that the production of 1kg of dried saffron requires about 80 kg of flowers of *C. sativus* [5] and 1 pound of saffron requires about 225,000 stigmas. Metabolic profiling of *C. sativus* has revealed more than 150 volatile compounds that contribute to the aroma, flavor, and color [6]. Nowadays, in the food industry, saffron is mostly used as a coloring agent and for food-grade flavoring. The phytochemical analysis of saffron has revealed various primary and secondary chemical constituents that are present in different concentrations in different parts of saffron. The flower of this plant contains various bioactive compounds with a wide range of medicinal properties [7]. The phytochemical investigation of this wonder plant confirmed the presence of several volatile as well as non-volatile compounds primarily found in stigmas. Apart from the presence of a large number of minerals, sugars, vitamins, and essential proteins, saffron possesses some very special bioactive safranal, picrocrocin, crocin, and crocetin as shown in Table 1 [8,9,10,11,12,13,14,15,16,17,18,19,20,21,22,23,24,25,26]. Other bioactive compounds found in the stigma include anthocyanins, carotene, lycopene, and glycoside crocins, all possessing a wide range of medicinal properties [27,28,29]. For instance, crocin is reported to possess various medicinal properties such as anti-inflammatory, anti-convulsant, radical scavenger capacity, anti-depressant, and anti-cancer ones, and augments learning and memory [30,31]. Studies reveal that crocin found in stigmas of saffron alone has anti-inflammatory and antioxidant properties [32]. Moreover, crocin and crocetin are also reported to suppress the aggregation of amyloid-β plaques [33,34]. Similarly, essential oils extracted from saffron contain safranal, a monoterpene aldehyde that also possesses several therapeutic properties—antioxidant, anti-hyperglycemic, anti-inflammatory, anxiolytic, and anti-seizure ones [35,36,37]. The current review aims to unravel the composition of bioactive compounds from saffron and their therapeutic values in human diseases. Besides the presence of biologically active compounds, there are various nutrients present in saffron that show a protective role against oxidative stress, harmful microbes, and cancerous ailments in animals [38], thereby signifying its role in the nutraceutical industry [39]. Further, we have summarized the beneficial effects of saffron in Figure 1.

## 2. The Primary and Secondary Metabolites Found in Saffron

The primary chemical constituents and their concentrations present in saffron are mentioned in Table 2. In addition, there are various secondary chemical constituents present in different parts of saffron that have beneficial roles in human life. These secondary chemical constituents include apocarotenoids, monoterpenoids, flavonoids, phenolic acids, and phytosterols.

### 2.1. The Apocarotenoids of Saffron

In saffron, apocarotenoid synthesis is a bio-oxidative process and is produced by the CCD enzyme (carotenoid cleavage dioxygenase)-mediated cleavage of carotenoids. Various reports indicate that the stigmas of *C. sativus* are the rich source of apocarotenoids, i.e., crocetin and crocin [48,49]. These two apocarotenoids play an essential role in enhancing the specific color known as a saffron color and are used by the food industry as a coloring agent [50,51]. It has been reported that crocetin plays a pivotal role in the eradication of cardiovascular diseases, various neuro-disorders, hypersensitive problems, oxidative stress, tumors, and inflammatory problems [38,52,53,54,55]. Besides this, saffron also contains some fat-soluble carotenoids like lycopene, phytoene, phytofluene, a-carotene, b-carotene, and zeaxanthin [56]. 

The glycosylation of crocetin leads to the formation of a biologically active hydrophilic carotenoid in saffron, which is known as crocin [57]. The α-crocin is a type of crocin that is mostly present in saffron and forms about 10% of its dry weight [58,59]. Reports suggested that the entire contents of crocin form about 6 to 16% dry weight of saffron, which depends on the type of cultivar, origination, and conditions under which its harvesting and processing takes place [60]. Crocin acts as a cellular antioxidative agent and hence reduces lipid peroxidation and maintains membrane integrity [39].

### 2.2. Saffron-Based Monoterpenoids

The other biologically active secondary phytochemical constituents are monoterpenoids, which include picrocrocin and safranal. These two monoterpenoids are synthesized through the degradation of zeaxanthin. Picrocrocin is very essential for flavor and a bitter taste, while safranal plays a pivotal role in increasing the specific aroma of saffron [61]. Picrocrocin is a monoterpene glycoside that acts as a precursor for safranal and is present as a main constituent in the essential oil of saffron [62,63]. Picrocin is mostly present in the stigma and the petals of *C. sativus*, which exhibits anti-cancerous and memory-enhancer properties. Besides this, picrocin is used against various diseases like menstrual disorder, cardiovascular disorder, depression, and Alzheimer’s disease [18,28]. However, it has been revealed that safranal is usually present in the stigma part of the flower of *C. sativus* [61] and has a direct effect on the central nervous system. It acts as an anti-depressant, and anti-convulsant, and also plays an essential role in reducing the withdrawal syndrome [64,65]. 

### 2.3. Saffron-Based Flavonoids

The flavonoids and their derivatives are considered the second most biologically active secondary phytochemical constituent present in the stigmas of *C. sativus*. There are various types of flavonoids and their derivatives present in various parts of *C. sativus* and these flavonoids show tremendous amounts of medicinal and nutraceutical properties. These flavonoids and their derivatives include vitexin, orientin, kaempferol, isoorientin, naringenin, astragalin, dihydrokaempferol, myricetin, quercetin, rhamnetin, and populin [10,12,27,38,62]. Moreover, saffron-based compounds such as crocin, safranal, picrocrocin, crocusatin D-I, isophorone, lycopene, and crocetin possess a wide range of therapeutic properties (Table 3, [66,67,68,69,70,71,72,73,74,75,76,77,78,79,80,81,82,83,84,85,86,87]).

### 2.4. Phenolic Acids in Saffron

The chemical analysis of saffron revealed various types of hydroxycinnamic acids, which include chlorogenic acid, caffeic acid, methylparaben, gallic acid, and pyrogallol [88]. Hydroxybenzoic acids act as the precursor for the biosynthesis of flavonoids and are reported from different parts of *C. sativus*. Hydroxycinnamic acids like h-coumaric acid, p-hydroxybenzoic acid, sinapic acid, and vanillic acid have been reported from the petals of saffron [89]. However, p-hydroxybenzoic and benzoic acids were also reported from the pollens of *C. sativus* [88].

### 2.5. Saffron Phytosterols

There are various types of phytosterols present in the different parts of saffron; however, phytosterols like β-sitosterol and stigmasterol were reported from both stigmas and petals while fucosterol was reported only from the petals of *C. sativus* [24,47]. 

## 3. Role of Saffron Bioactive Compounds against Human Diseases

As per the reports of the World Health Organization (WHO), human beings are paying most of their attention to the use of herbal medicines as they have the least side effects [90,91]. A huge number of medicinal plants are currently being investigated to explore natural products as the best alternative to unsafe drugs [92,93,94]. Regardless of the growth in recent diagnostics and treatment strategies, the prevalence of diseases at the global level is still rising. Although a wide range of therapeutic strategies are satisfactory in treating a large number of diseases, these treatments are failing in many cases such as cancers, cardiovascular diseases, etc., and the worst part of these statements lies in their severe health-complicating side effects. Against this backdrop, scientists are committed to exploring natural therapeutic agents as novel ways to treat a wide range of diseases. 

The chemical analysis has reported the presence of about nearly 150 non-volatile and volatile components in saffron. The crocetin, picrocrocin, and crocin are non-volatile and the terpene, terpenoids, and safranal are the volatile components. Among the volatile components, safranal has various therapeutic applications [37]. José Bagur et al. [95] reported that picrocrocin, safranal, and crocin play an essential role in enhancing the bitter taste, aroma, and different colors in saffron, respectively. The different bioactive components associated with different parts of the *C. sativus* plant have various medicinal properties, which depend on their unique chemical structure. Tong, Y., et al. reported that the highly studied bioactive components like safranal, crocin, and picrocrocin of saffron play an antagonistic role against various diseases like Alzheimer’s, diabetes, cancer, cardiovascular disease, and erectile dysfunction [96]. A large number of investigations reveal that saffron-based bioactive compounds help to act as an anti-metabolic syndrome, antioxidant, memory enhancer, cardioprotective compound, anti-inflammatory compound, anti-depressant, and anti-cancer agent [97,98,99,100]. The following sections of this review are aimed at unraveling the potential of saffron as the source of novel molecules to treat major human diseases.

### 3.1. Role in the Treatment of Cancers

**Figure 2:**
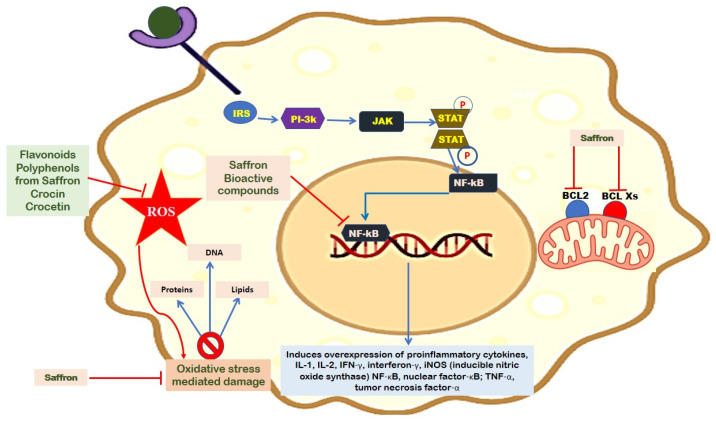
A schematic illustration shows the inhibitory function of saffron which leads to decrease in the production of reactive oxygen species, IL-17, TNF-α, NF-κB, IL-1β, and NO. The figure also demonstrates that saffron helps in the suppression of apoptosis by inhibiting the function of BCL-2.

Saffron-based bioactive compounds are reported to exert anti-cancer effects through the induction of apoptosis, regulation of the immune response, and anti-inflammatory effects. For instance, crocin extracted from saffron was reported to exert anti-cancer effects in colorectal cancer cell lines [101]. Kawabata et al. [102] reported similar results against colitis-associated colon carcinogenesis. The authors further showed that crocin inhibited cancers by downregulating the transcription of cytokines such as tumor necrosis factor-α [TNF-α], nuclear factor-κB [NF-κB], interleukin-6 [IL-6], interferon-γ, COX-2, IL-1β, and inducible NO synthase [iNOS] (Figure 2). 

Moreover, crocetin treatment inhibited colon cancer by downregulating the levels of vascular endothelial growth factor (VEGF) matrix metalloproteinase-9 and NF-κB [103]. Crocetin was also reported to exert anti-cancer effects on human gastric cancer cells (BGC-823 cells), further validating the therapeutic role of saffron [104]. 

Further, in vivo studies carried out in rats revealed that saffron functions as a chemoprotective agent by enhancing the caspase-3 cleavage, inhibiting the activity of nuclear factor-κB (NF-κB), and leading to cell cycle arrest [105]. Other studies reported the role of crocin in inhibiting liver cancers by inhibiting the interleukin (IL)-6/STAT3 cascade [106]. For instance, crocetin administration inhibited the growth of proliferating MIA-PaCa-2 cells. Similarly, it was reported that crocin inhibited pancreatic cancer cells [107]. Thus, the current literature strongly suggests that saffron is a source of promising therapeutic agents such as crocin and crocetin employed against several cancers [108]. Consequently, saffron needs special attention by employing pre-clinical investigations to further validate its anti-cancer effects and to troubleshoot the optimum dosage for effective treatments.

### 3.2. The Defense to Cardiovascular Diseases (CVDs)

CVDs are caused due to dietary and metabolic imbalances affecting heart muscles and the blood circulatory system, resulting in impairment of blood supply to the brain, heart, and other organs. Data reveal that CVDs are the leading cause of human deaths on the global scale [109]. Natural compounds have played a critical role in controlling the progression and development of CVDs and are the best candidates to cure metabolic disorders related to the circulatory system [110]. For instance, crocin, a bioactive constituent in saffron, is reported to regulate normal functions of the cardiovascular system by reducing dyslipidemia and oxidative stress [110]. Similarly, Chahine, et al. [111] reported that saffron bioactive compounds improved the free radical scavenging activity in rabbits due to cardiac dysfunctioning mediated by doxorubicin. The presence of crocin in saffron leads to a decrease in the level of triacylglycerol and cholesterol in plasma, thus aiding in the control of cardiovascular complications [112]. Safranal and crocin can eliminate ROS, thus helping to prevent cancers and cardiovascular diseases [41]. For instance, reports suggest that saffron tea, having lycopene, helps in reducing the risk of CVD development [86]. Similarly, crocin and crocetin were found to activate vasoconstriction during hypertension and were also found to treat endothelial dysfunction and reduce aortic contraction problems [113]. 

One of the best pharmaceutical effects of saffron is its ability to reduce the levels of serum triglycerides, very low-density lipoprotein (VLDL), low-density lipoprotein (LDL), and cholesterol, hence creating low levels of fat and cholesterol deposition, thus reducing the risk of CVDs like atherosclerosis, coronary artery disease, and hypertriglyceridemia [114]. Moreover, crocetin was also reported to have an anti-atherosclerotic effect in model rabbits with atherosclerosis [115]. Similarly, crocetin supplementation reduced the accumulation of oxidatively modified low-density lipoprotein (Ox-LDL) and lessened the formation of atherosclerotic lesions [116]. Previously, it was reported that crocin decreased the expression of Lectin-like oxidized LDL receptor 1 (LOX-1) and nuclear factor kappa B (NF-κB), which in turn reduced the progression of coronary artery disease (CAD). Overall, these reports suggest that saffron-derived compounds have great potential to reduce the negative effects of CADs and regular consumers of saffron-based foods may be protected against the clinical implications of CADs [86,117,118]. Additionally, saffron may serve as a strong functional food or nutraceutical for health as well as commercial purposes. 

### 3.3. Antioxidant and Anti-Inflammatory Properties of Saffron Bioactive Compounds

Antioxidant compounds are very critical to defend against cancers, metabolic disorders, and aging by inhibiting the generation of ROS. Metabolites such as picrocrocin, crocin, and safranal derived from saffron are identified as potential antioxidants to scavenge the ROS [119]. Crocin helps to protect the organs such as the liver, kidneys, and brain from damage induced by oxidative stress [74]. Inflammation in animals may cause severe damage to organ systems, thus leading to functioning impairments [120]. For instance, studies revealed that saffron-based metabolites such as kaempferol, crocetin, crocins, and quercetin are found to inhibit the generation of pro-inflammatory cytokines to prevent damage induced by chronic inflammation [121].

Crocins found in saffron are reported to be a potent anti-inflammatory compound [122]. For instance, the hyperglycemic effect, activation of protein kinase C (PKC), and elevation of ROS trigger the activation and import of NF-κB to induce the expression and coding of pro-inflammatory factors like IL-6, IL-1b, and TNF-a [55]. In this context, in macrophages, the crocin inhibits enzymes such as cyclooxygenase-1 (COX1) and cyclooxygenase-2 (COX2) and inhibits the NF-κB to block the production of prostaglandin-2 (PGE2), hence playing a critical role in the inhibition of inflammatory responses [123].

### 3.4. Anti-Diabetic Effects of Saffron

Crocin, safranal, and crocetin are the main components found in saffron, which are reported to possess anti-diabetic activity [124]. Studies back up the role of saffron-based metabolites acting as antioxidants to reduce oxidative stress and also negate the effects of hyperglycemia [33]. In an experiment, it was reported that in obese prediabetic individuals, the oral administration of saffron improved the glycemic indices and antioxidant activity [125]. In another study, saffron was found to enhance the uptake of glucose and inhibit the activity of protein tyrosine phosphatase 1B (PTP1B), an inhibitor of the insulin signaling pathway [126]. Similarly, crocin helps in reducing levels of glucose, TG, HbA1c, total cholesterol, IL-1b, IL-2, IL-4, IL-10, and NF-kB and upregulates the synthesis of enzymes such as nuclear respiratory factor 2, manganese superoxide dismutase 1, and catalase (CAT) and heme oxygenase-1 [127]. Moreover, the crocin was found to decrease the ratio of Bax/Bcl-2 to diminish the myocardial apoptosis and additionally increase the AMPK phosphorylation to normalize the autophagy dysfunction in case of a diabetic myocardium. The crocin also inhibits apoptosis by reducing the levels of P53 in cells [128]. In the same way, the administration of saffron to endothelial progenitor cells decreased apoptosis, reduced the caspase 3 levels, and reduced the progression of diabetes by affecting the PI3K/AKT-eNOS and ROS pathways [71].

### 3.5. Role in Decreasing the Progression of Neurological Disorders

For decades, neurodegenerative diseases have proven to be a significant threat to human health due to the low availability of drugs [129]. Currently, a large number of plants have been explored to screen natural products as the best alternatives to synthetic drugs [130]. Saffron is employed in several studies to prove its potential to treat neurological diseases. Since very limited drugs are available to treat neurological diseases, saffron may be the ray of hope against several neurological diseases, such as AD, anxiety, depression, Parkinson’s disease, etc. The following reports further highlight the role of saffron and derived compounds in decreasing the progression of neurological disorders. For instance, considerable studies demonstrate that saffron-based bioactive compounds have a positive impact on the functioning of cognition and memory in animal models [98,131,132]. In addition, crocin and crocetin were reported to help in neuroprotection by enhancing the viability of cells, suppressing apoptosis as well as the production of ROS, increasing the expression of protein kinase B, activating mTOR, and activating MAPK phosphorylation [133]. 

The saffron extracts were found to aid in the clearance of Aβs in Alzheimer’s disease models by the upregulation of LRP1 and P-gp [134]. Crocin and crocetin were reported to inhibit LPS-induced NO release, inhibit NF-κB activity, and repress the production of ROS, TNF-α, and IL-1β [67]. The crocin is believed to increase the levels of glutathione peroxidase (GPx) and superoxide dismutase (SOD), which in turn decreases the formation of advanced glycation end products (AGEs) and suppresses ROS production in serum and brain tissues of mice models [133,135]. Saffron-identified compounds such as crocin, flavonoids, saponins, and tannins play a critical role in alleviating the clinical features of AD, due to anti-inflammatory and antioxidative properties [70,136]. The antioxidant effect is primarily displayed by the crocin-induced expression of the γ-glutamyl cysteinyl synthase (γ-GCS) gene, which in turn leads to the synthesis of glutathione (GSH), a strong antioxidant molecule [70]. Both crocetin and crocin are reported to decline in the accumulation of Aβ to enhance neuroprotection in patients with AD [137,138]. Other therapeutic effects of crocin include a significant decline in hippocampal ROS, induction of enhanced activity of superoxide dismutase (SOD), and role in attenuating DNA fragmentation and apoptosis [72]. Pre-clinical reports suggest that saffron extracts impart neuroprotective effects via the regulation of phosphatidylinositol 3-kinase (PI3K) and mitogen-activated protein kinase (MAPK) involved in the survival of nervous tissues [80]. Studies also demonstrated the role of saffron extracts in alleviating anxiety and depression in patients with AD [139]. 

Parkinson’s disease is another neurodegenerative disease still awaiting precise treatment at the global level. This disorder is associated with the formation of Lewy bodies and an ill defense against oxidative stress by dopamine-secreting neurons, leading to the degeneration of neurons. Hence, there is a lack of functioning of dopaminergic neurons in the substantia nigra limbic system [140]. Reports suggest that crocin plays a critical role in stimulating the mTOR signaling, leading in turn to a decreased activation of caspase-9, glycogen synthase kinase-3β (GSK-3β), and forkhead box transcription factor of the O class (FoxO3a) [66]. In addition, crocin leads to an enhanced expression of miRNA-221 and microRNA-7 (miRNA-7), both responsible for the activation of Akt/mTOR and also aiding in the relief of α-synuclein formation and the inhibition of apoptosis [66]. Experiments demonstrated neuroprotective effects of crocin in PD-induced mouse models by decreasing oxidative stress [83]. In PD rat models, improved spatial memory was observed upon treatment with saffron extracts [141]. The saffron extracts were reported to enhance the cellular expression of nuclear factor erythroid 2–related factor 2 (Nrf2), mediating resistance to oxidative stress in rotenone-induced PD models [142]. Inoue E. et al. [143] reported that saffron constituents such as crocin-1, crocin-2, and crocetin play a pivotal role in the inhibition of α-synuclein and Lewy body accumulation.

Huntington disease (HT) is another deadly neurological disorder, characterized by several clinical features such as emotional problems, uncontrolled movements, and loss of thinking ability (cognition), resulting in physical, psychological, and behavioral changes [144]. The genetic basis of HT involves the incorporation of multiple repetitions of cytosine–adenine–guanine (CAG) trinucleotides in the Huntington (HTT) gene, resulting in the synthesis of an abnormally long Huntington protein, which is further cut into smaller fragments, which in turn aggregates together and accumulates in nerve cells [144]. The aggregation and accumulation of these fragments both intracellular and extracellular to neurons results in the impairment of nerve functions; hence, the longer-run outcome is neurodegeneration [145]. The saffron extracts were shown to diminish the modulations caused by 3-Nitropropionic acid (3-NP), resulting in lessening effects of clinical features in Huntington models of mice [81]. Fotoohi et al. [81] also demonstrated that saffron decreases the glutathione (GSH), catalase, and SOD activities and on the other hand prevents an increase in the levels of malondialdehyde (MDA) and nitrite oxide. Zhang et al. [146] also reported that crocin led to enhanced levels of GSHPx, GSH, and SOD and decreased the levels of glutathione disulfide (GSSG) and MDA. Therefore, saffron displays considerable potential to reduce the progression of neurological disorders and this wonder plant needs further attention in terms of research as well as clinical significance. Further well-designed clinical trials are needed to validate the potential benefits of saffron in neurodegenerative diseases and to determine the optimal dosage, treatment duration, and safety profile.

## 4. Antimicrobial Activities of Saffron

Antimicrobial resistance to traditional antibiotics and its speedy progress have raised serious alarm in the management of infectious diseases across the globe. Numerous studies have been conducted recently with the goal of identifying viable alternatives to combat microbial infections and the antimicrobial resistance threat. One such promising area is harnessing the potential of phytochemical molecules for the development of plant-based drugs. Antimicrobials produced from plants are favored because of their desirable safety and effectiveness profiles. Plant metabolites function by selectively attacking microbial cell membranes; obstructing the production of microbial DNA, RNA, and enzymes; and causing disruptions to the expression of efflux pumps and quorum sensing. Additionally, they boost the antibacterial properties of conventional antibiotics by working in concert with them [147]. Many studies have shown that phytochemicals have great antimicrobial potential with several mechanisms of action against both susceptible and resistant microorganisms [148]. Similarly, it is evident from earlier research that *C. sativus* petals possess strong antibacterial, antifungal, and antioxidant activity [149,150]. A notable antibacterial activity of saffron petals’ methanolic extract was reported by Asgarpanaha et al. [151] against *Escherichia coli*, *Bacillus cereus*, *Salmonella typhi*, *Staphylococcus aureus*, and *Shigella dysenteriae.* Similarly, another study has revealed that saffron extracts showed significant antibacterial activity against *P. aeruginosa* and *S. aureus* [152]. Recently, it was reported that saffron petal extracts showed an antibacterial effect against food-borne pathogens such as *C. perfringens*, *C. difficile*, and *C. botulinum*, which further highlights its role as a valuable natural alternative source to conventional preservatives [150]. In addition to antibacterial activity, saffron extracts also show potent antifungal activity against different fungal pathogens such as *Aspergillus fumigatus*, *Candida albicans*, and *Aspergillus niger* [153]. The presences of safranal and crocin have been reported to be the main drivers of antimicrobial and antifungal activity of saffron extracts. However, future studies should further explore the metabolic diversity of saffron with an aim of finding novel drug molecules with antimicrobial potential and that can be viable resources for combating the antimicrobial resistance threat sustainably. 

## 5. Nutritional Benefits of Saffron

Plants are necessary for providing humans with everyday needs like food, shelter, fiber, and medicinal uses. Plants with medicinal and nutritional benefits are key for resources for food and therapeutic usage, especially in developing regions. At present, natural food intake is becoming more and more popular as opposed to processed foods with artificial ingredients. Due to the presence of numerous significant bioactive components, such as crocin (which gives saffron its color), safranal (which gives saffron its smell), and picrocrocin (which gives saffron its taste), the dried stigma of *C. sativus* is used extensively in cooking as a flavoring and dietary spice. *C. sativus* possesses huge nutritional benefits in addition to its medicinal properties. It contains proteins and amino acids (12%); minerals (5%); lipids (5%); fibers (5%); various sugars (63%); vitamins [(B1 (riboflavin), B2 (thiamine)]; and different macro- and micronutrients like Ca, K, Mg, P, and Fe [154,155]. Based on available data, we show the nutritional value of *C. sativus* in Table 2. Owing to its high nutritional importance, it is important to use different omics and metabolic engineering tools to further improve its nutrient components as well as its production. 

## 6. Role of Omics and Metabolic Engineering for Improving Saffron Bioactive Compounds 

**Figure 3:**
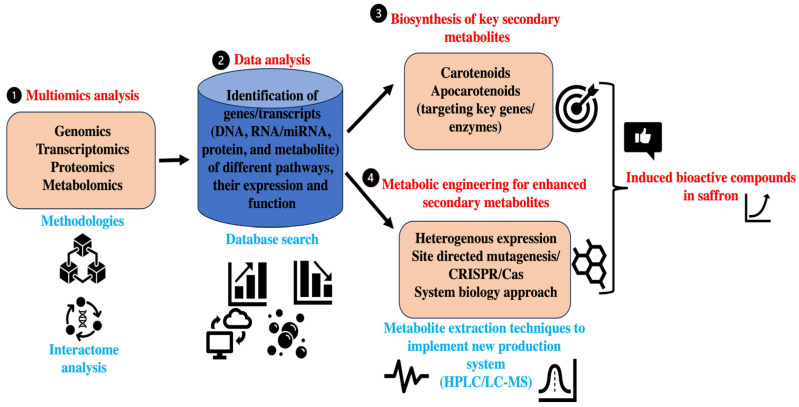
An illustration of the role of omics and metabolic engineering (CRISPR/Cas) for increasing the production of potential bioactive compounds in saffron.

The application and integration of other omics, such as genomics, proteomics, and metabolomics, can give more insights on the functional attributes of saffron bioactive substances and their biochemical pathways. In the past, a multiomics approach has been an excellent platform to unravel the role of different plant metabolites by integrating the interpretation of vast data from biological processes at the gene, protein, and metabolite levels along with other in silico and analytical analyses. Genome-scale enzyme and pathway annotations and omics technologies have revolutionized research to decrypt plant metabolism and produced a growing list of functionally characterized metabolic genes and pathways in both model and crop plants. Also, advances in metabolic route mapping infrastructure and omics methods make it easier to uncover new pathways in a wide range of plant species [156]. Similarly, transcriptomics can provide a snapshot of the expression profiling of metabolic pathway genes, transcription factors, and their regulation. On the other hand, genome-wide association studies (GWASs) have been frequently used to elucidate the genomic architectures underpinning the phenotypes of interest. Metabolite GWASs (mGWASs) can identify a variety of genes related to metabolic traits, including transcription factors and biosynthetic genes [157]. Therefore, the application of the above-mentioned molecular approaches like omics and metabolite GWASs in saffron can provide novel insights on identifying genes related to metabolic traits. At present, the isolation and identification of key plant bioactive compounds cannot be produced profitably by comprehensive chemical synthesis. As a result, their industrial supply procedures mostly involve extracting end products or precursors from plant resources. In this regard, biotechnological manufacture has led to several benchmark studies on producing key plant-originated bioactive molecules in heterologous hosts. Similarly, a heterologous host system can be used for the production of saffron bioactive compounds for larger-scale production. Crocetin and crocin biosynthesis has received significant attention because of its high scientific and economic interest, and attempts to metabolically engineer their process in different microbial systems have been described with limited success. Therefore, new tools like gene editing are required to further accelerate the metabolic engineering in saffron for bioactive molecule production. In recent years, synthetic biology and metabolic engineering have advanced at a rapid pace, thereby offering new ways for improving targeted metabolite production. One of the key applications of these tools is the building of cell factories to produce high-value-added bioactive compounds [158]. Recently, the CRISPR/Cas tool kit has been commonly used for efficient metabolic engineering in heterogeneous hosts for the production of desired bioactive compounds. Similarly, approaches can be used for producing saffron bioactive compounds like crocin, safranin, etc. However, this will require an in-depth understanding of the metabolic pathways and their encoding gene networks related to different bioactive molecules in saffron. In this regard, the use of multiomics in saffron can help in identifying target genes, transcriptional factors, and proteins that regulate metabolite production (Figure 3). This will also play a key role in metabolic engineering in saffron as well as its heterologous model systems. In comparison to other plants, there have been no investigations on the function of multiomics and metabolic engineering in saffron bioactive chemical synthesis. Because of its economic relevance and demand, it is critical to understand the molecular complexity of saffron metabolic pathways and improve its metabolic traits. Keeping in consideration the critical therapeutic values elucidated by in vitro/in vivo studies using raw extracts in animal cell lines and animal models, the red gold plant (saffron) demands serious attention in terms of medicinal values (Table 4). Also, the impact of climatic changes on saffron cultivation is necessary to develop resilient saffron cultivars to survive under unfavorable conditions.

## 7. Regulation of Gut Microbiome by Saffron and its Metabolites: A Concise Account

The human gut harbors many microbial species including fungi, bacteria, archaea, protozoa, and viruses [171]. The existence of microbiomes and their metabolic products plays a critical role in the versatile functions of the gut, which include digestion and absorption and immunity of the gut to pathogenic microbes, and also induces cell proliferation and differentiation. For instance, metabolic products mediate the balance of Treg cells and Th17 cells to regulate inflammation [172]. Gut microbiota can also assist the host by converting dietary nutrients into bioactive metabolites like fermenting nondigestible carbohydrates into short-chain fatty acids (SCFAs), which are the primary metabolites of intestinal microbiota, including butyrate, propionate, and acetate. Recent research indicates that SCFAs, particularly butyrate, have crucial intestine and health-modulating activities. Hence, gut microbiota has a vital function in enhancing host health. Despite a huge number of drugs being designed to treat numerous diseases, the adverse effects such as decreasing the beneficial gut microbiome, risk of infection, toxicity, and malignancies diminish their applicability in humans. Saffron and its derivative compounds are alternatives to eliminating the pro-inflammatory bacteria and regulating the beneficial microbes such as Firmicutes/Bacteroides in mouse models [173]. These studies also demonstrated the role of saffron in regulating microbiome-associated metabolites such as 2 hydroxyglutaric acid, cholesterol, uric acid, 2-hydroxyhexanoic acid, and allantoic acid. In conclusion, saffron plays a pivotal role in maintaining the homeostatic balance in regulating the gut microbiome and healthy state of the gut [173]. Recently, Pontifex et al. [174] reported that the saffron extract reduces anxiety-related behavior in a rat model of low-grade inflammation via modulating the microbiota and gut metabolites. Similarly, in mice, Crocin-I modulates gut microbiota and intestinal inflammation to protect against high-fat-diet-induced obesity. These studies further provide the evidence that saffron plays an important role in modulating gut microbiota and disease treatment in different animal models. These studies necessitate future studies on exploring different saffron bioactive compounds for shaping the gut microbiome, which can have a beneficial impact on human health.

## 8. Conclusions and Future Directions

The saffron plant is a repository of nutritionally and therapeutically important bioactive compounds. The bioactive constituents improve the antioxidant defense, anti-inflammatory effect, and anti-diabetic effect, and impede the progression of neurodegenerative diseases. However, very limited knowledge is available regarding the activities of saffron in almost all diseases. We strongly believe our current compilation offers a strong up-to-date account of the medicinal applications of saffron and its critical bioactive compounds. Further, the state-of-the-art knowledge regarding this wonderful cash crop will aid in promoting the future research investigation and exploration of saffron-based compounds as therapeutic tools for human ailments. For the elucidation of detailed mechanisms, a further exploration of pathophysiological and molecular experiments is required to validate the therapeutic potential of saffron. However, to elucidate more detailed mechanisms, further studies, especially molecular and pathological experiments warrants future investigation. Still, clinical trials with exclusive results are critical to validate the pharmacological effects of saffron-based compounds. Owing to its multiple benefits, the demand for saffron is rising; therefore, to fulfill market demand, there is a need to use different metabolic engineering tools like CRISPR/Cas for augmenting its metabolite production. Furthermore, as environmental stressors drastically impact *C. sativus* yield and quality, therefore creating climatically tolerant cultivars is essential to meet future demand.

## Figures and Tables

**Table 1:** Secondary phytochemical constituents present in different parts of saffron and their medicinal properties.

Name of Compound	Present in Plant Part	Medicinal Properties	References
Vitexin	Leaves	Antioxidative, anti-cancerous, anti-inflammatory, anti-hyperalgesic, neuroprotective	[9,10]
Orientin	Tepals	Anti-viral, antibacterial, anti-depressant, cardioprotective, anti-aging, and antioxidant	[10,11]
Kaempferol	Tepals	Reduces the risk of cancer and metastasis Anti-depressive Inflammation Antioxidant	[12,13]
Isoorientin	Tepals	Prevents liver damage; antioxidative, anti-inflammatory, and anti-nociceptive properties	[10,14]
Naringenin	Petals	Antimicrobial, anti-mutagenic, and anti-cancer; reduces cardiovascular and gastrointestinal diseases	[15]
Astragalin	Stigmas	Anti-obesity, anti-diabetic, anti-ulcer, anti-osteoporotic	[16]
Dihydrokaempferol	Petals	Used as a treatment for rheumatoid arthritis (RA)	[17,18]
Myricetin	Stigmas	Plays role in the scavenging of ROS; anti-allergic, anti-platelet aggregation, and anti-hypertensive	[19]
Quercetin	Flowers	Reduces blood sugar level and reactive oxygen species and prevents heart diseases	[10]
Rhamnetin	Stamens and petals	Plays role in attenuation of melanogenesis by reducing oxidative stress and pro-inflammatory mediators	[20]
Populin	Stigmas	Anti-diabetic, reduces the activity of aldose reductase enzyme, and antioxidant	[12]
**Phenolic Acids present in Saffron and their medicinal properties**			
Chlorogenic acid	Stigmas	Prevents weight gain, reduces development of liver steatosis, and reduces lipid accumulation in liver and enhances insulin sensitivity	[21]
Caffeic acid	Stigmas	Anti-viral especially against herpes and HIV virus, and treatment for cancer	[19]
Methylparaben	Stigmas	Antibacterial; antifungal	[19,22]
Gallic acid	Stigmas	Antioxidative, antineoplastic, and anti-inflammatory; useful for cardiovascular disorders	[19,22]
Pyrogallol	Petals	Antibacterial; antifungal; and anti-malarial	[22,23]
**Phytosterols present in saffron and their medicinal properties**			
β-Sitosterol	Flowers (stigma, petals, and pollens)	Reduces cholesterol levels; treatment for heart disease and rheumatoid arthritis (RA)	[24]
Stigmasterol	Flowers Stigmas Stamen Perianth corms	Used as a drug for cancer therapy by inducing intracellular signaling pathways in various types of cancers. It affects the pathways of gastric and ovarian cancers; these pathways include Akt/mTOR and JAK/STAT	[24,25]
Fucosterol	Flowers (petals)	Anti-cancerous, anti-diabetic, antifungal, anti-osteoporotic, and anti-cholinergic; reduces blood cholesterol level	[24,26]

**Table 2:** The primary constituents and their concentrations reported in saffron plants.

**Chemical Constituents**	**Concentration (%)**	**References**
Moisture	10–14	[40,41]
Ash	06–07	
Crude fat	05–08	
Crude protein	12–14	
Crude fiber	04–05	
Nitrogen-free extract (NFE)	52–63	
	NFE	
Reducing sugars	20	[40,41,42]
Gums and dextrins	09–10	
Starch	06–07	
Pentoses	06–07	
Different mineral nutrients and their concentrations (mg/g) present in the stigmas of *C. sativus*		
**Minerals**	**Concentration (mg/g)**	**References**
Phosphorous	3770	[43,44]
Magnesium	1350	
Calcium	1070	
Iron	110	
Potassium	14.86	
Sodium	100	
Different vitamins and their concentrations (mg) present in the stigmas of *C. sativus*		
**Vitamins**	**Concentrations (mg)**	**References**
Vitamin-A	27	[22,45,46]
Vitamin-B1	0.115	
Vitamin-B2	13	
Vitamin-B6	1.01	
Vitamin-C	80	
Different concentrations of different fatty acids (g/100g) in saffron based on GC analysis		
**Fatty Acids**	**Concentrations (g/100g)**	**References**
Palmitic acid	16.2	[24,47]
Linoleic acid	28.5	
Linolenic acid	21.0	
Stearic acid	-	
Oleic acid	-	
Arachidonic acid	-	

**Table 3:** The biological activities of saffron-based metabolites, which are used as potential therapeutic agents against a wide range of diseases.

Name of Metabolite	Biological Activities	References
Crocin	Anti-schizophrenia, Antifatigue, Anti-Alzheimer’s, Neuroprotective, Anti-depressant, Anti-diabetic, Therapeutics for multiple sclerosis, Antioxidative, Anti-tumor, Therapeutics for neuro-retinal diseases, Anti-inflammatory, Anti-apoptotic, Mitigates blood pressure and heart rate	[66,67,68,69,70,71,72,73,74,75,76,77,78]
Safranal	Anti-convulsant Anti-Alzheimer’s Anti-Huntington disease Therapeutics for retinal degeneration Antioxidative Prevention of respiratory distress	[33,79,80,81,82]
Picrocrocin	Antiproliferative	[83]
Picrocrocin	Anti-cancer activity	[84]
Crocusatin D, Crocusatin F, Crocusatin G, Crocusatin H, Crocusatin E, Crocusatin I	Withdrawal syndrome, depression, spatial memory	[85]
Isophorone	Hyperglycemia–glucose uptake/metabolism Parkinson’s disease	[85]
Lycopene	Reduces effects of CVD	[86]
Crocetin	Anti-Parkinson’s disease Protection of heart diseases Anti-apoptotic	[55,87]

**Table 4:** Therapeutic values, dose, and host applicability of saffron extracts and isolated compounds used under in vivo/in vitro conditions.

Name of Saffron-Based Compound	The Experimental Animal/Cell Line	Effective Dose of the Extract	Disease Treatment under Tnvestigation	References
Crocin	Adult male Wistar rats	15 and 30 mg/kg/day	Learning and memory impairment, chronic stress	[74]
	Male Wistar albino rats	5, 10, and 20 mg/kg/day	Cardioprotective effect, alleviating oxidative stress	[159]
	Male Wistar rats	50, 100, and 200 mg/kg/day	Aids in prevention in the reduction in platelet counts	[159]
	Adult male mice	35 days, 20 mg/100 g	To neutralize hemolytic anemia	[160,161]
	Rats	5, 10, and 20 mg/kg/day	Cardioprotective	[159]
	Adult male Wistar rats	25 and 50 mg/kg	Alleviating oxidative stress	[162]
Crocetin	Male Wistar rats	40 mg/kg for weeks	To prevent the development of insulin resistance	[163]
	Cultured retinal ganglion cells	3 mM	Alleviating oxidative stress	[164]
	Male Wistar rats	50, 100, and 200 mg/kg	Alleviating oxidative stress	[163]
	Male Wistar rats	1–50 mM	Neuroprotection	[165]
	Male Wistar rats	40 mg/kg for 8 weeks	To prevent the development of insulin resistance	[163]
	Mice	50–600 mg/kg	Anti-depressant	[166]
Safranal	Male Wistar rats	0.025, 0.05, and 0.1 mL/kg	Alleviating oxidative stress	[160]
	Mice	0.15–0.5 mL/kg	Anti-depressant	[166]
	Adult male NMRI rats	24 h for 72 h, 727.5 mg/kg	Prevents oxidative damage in hippocampal tissue from ischemic rats	[167]
Saffron tablets	Human volunteers in placebo study	400 mg/day	Treatment of depression/anxiety and safety evaluation	[168]
	Human volunteers in pilot study	200 mg	To alleviate erectile dysfunction and infertility	[169]
	Human volunteers in pilot study	30 mg/day (15 mg twice a day)	Anti-depressant	[170]

## Data Availability

Not applicable.
